# Specificity of UV-C LED disinfection efficacy for three N95 respirators

**DOI:** 10.1038/s41598-021-94810-4

**Published:** 2021-07-28

**Authors:** C. Carolina Ontiveros, David C. Shoults, Sean MacIsaac, Kyle D. Rauch, Crystal L. Sweeney, Amina K. Stoddart, Graham A. Gagnon

**Affiliations:** Centre for Water Resources and Studies, Department of Civil and Resource Engineering, Dalhousie University, 1360 Barrington St, Halifax, NS B3H 4R2 Canada

**Keywords:** Engineering, Materials science, Lasers, LEDs and light sources, Microbiology techniques

## Abstract

The recent surge in the use of UV technology for personal protective equipment (PPE) has created a unique learning opportunity for the UV industry to deepen surface disinfection knowledge, especially on surfaces with complex geometries, such as the N95 filter facepiece respirators (FFR). The work outlined in this study addresses the interconnectedness of independent variables (e.g., UV Fluence, respirator material) that require consideration when assessing UV light efficacy for disinfecting respirators. Through electron microscopy and Fourier-transform infrared (FTIR) spectroscopy, we characterized respirator filter layers and revealed that polymer type affects disinfection efficacy. Specifically, FFR layers made from polypropylene (PP) (hydrophobic in nature) resulted in higher disinfection efficiency than layers composed of polyethylene terephthalate (PET-P) (hygroscopic in nature). An analysis of elastic band materials on the respirators indicated that silicone rubber-based bands achieved higher disinfection efficiency than PET-P bands and have a woven, fabric-like texture. While there is a strong desire to repurpose respirators, through this work we demonstrated that the design of an appropriate UV system is essential and that only respirators meeting specific design criteria may be reasonable for repurposing via UV disinfection.

## Introduction

There has been acute shortages of single-use N95 filter facepiece respirators (FFR) during the COVID-19 pandemic^1^. Consequently, an urgent call was made for suitable disinfection technologies for FFR reuse in response to shortages to overcome the critical need for PPE to ensure healthcare staff safety^2^. Some healthcare jurisdictions resorted to improvised FFR recycling programs where entire rooms were designated as spaces to expose used FFR to ultraviolet (UV) light to disinfect them for reuse^3^. The efficacy and suitability of several disinfection technologies have been investigated in recent years, including autoclaving, ethylene oxide (EtO), vaporized hydrogen peroxide (VHP), bleach, microwave irradiation, and UV irradiation^4,5^. One study concluded that FFR disinfection by UV-C light holds several advantages over microwave oven and bleach due to melting of the respirator material and lingering smells of bleach, respectively^5^.

UV irradiation has been used in drinking water and wastewater treatment for decades, and more recently, has been shown to be effective for surface disinfection in food processing^6–8^ and on other surfaces^9,10^. UV disinfection has several advantages over other disinfection methods due to its generally low environmental impacts, ease of use, and relatively small space requirements^11^. The suitability of UV disinfection as a germicidal process for more intricate materials (such as FFRs) is less understood. One of the limitations of UV treatment technologies is its ineffectiveness when light-shielding materials are present. UV shielding is particularly important when considering FFRs, as their surface macro-geometry is complex and may lead to shaded areas that are not exposed to UV light. The micro-geometry of FFRs is also complex as it consists of a complex matrix of woven fibers that can shade inoculum once they penetrate into mask layers. FFR geometry and material properties are hypothesized to play a role in shielding organisms from UV, resulting in reduced efficacy^5^. Thus, there is a knowledge gap in understanding the interactions between UV light and complex surfaces and a need for further investigation to harness UV disinfection technologies for decontaminating FFR materials.

Before the COVID-19 pandemic, data on UV disinfection of FFR was limited^12^. Previously conducted studies examining UV irradiation on FFRs are not current, contained methods that were difficult to compare with other studies, and required an update to fit the needs of the COVID-19 pandemic^5,13–15^. Supply chain interruptions for personal protective equipment (PPE) led some jurisdictions to authorize emergency reuse of FFRs and other PPE, which resulted in an increase in studies investigating the efficacy of UV disinfection of these materials^2,12,16,17^. A recent surge of surface-UV exposure products brought to market for disinfecting FFRs, personal objects, and surfaces have resulted in additional measures by the U.S. Food and Drug Administration (FDA) to ensure consumer safety^18,19^. However, the guidelines provided by the FDA and the National Institute of Standards and Technology (NIST) are in their preliminary stage, and there is a lack of guidance towards best practices for validating FFR disinfection processes.

The design of UV disinfection studies for FFRs requires careful consideration of several variables that may impact results. For example, the FFR model used, material layers selected for inoculation, and microorganism loading density must be considered. Further, in light of the public health significance of N95 respirators, disinfection studies must be carefully designed to minimize the number of respirators needed for research purposes. Additionally, there has been use of both surgical and non-surgical N95 masks within hospital settings as a result of PPE shortages. Surgical N95 FFR are designed for slash and fluid protection and are also given FDA approval, while non-surgical N95 FFR provide filtration protection but must be coupled with a face shield in order to be safe from fluid exposure^20^. Accordingly, we designed an efficient experimental study with the aim of understanding the impact of multiple parameters on FFR material disinfection using UV irradiation. Experimental factors studied included UV fluence, test organism, inoculation concentration, inter-layer inoculation, and strap material. We also addressed the impact that FFR model type has on disinfection efficiency, which to date has not been sufficiently addressed in current literature.

## Methods

### Microbiological propagation and enumeration

All experiments were conducted with *Pseudomonas aeruginosa* PA01 and MS2 bacteriophage (ATCC 15597-B1) as performance surrogates for bacteria and viruses, respectively. For bacterial testing, tryptic soy broth (TSB) was inoculated with 100 µL of overnight culture to create a 4-h subculture. *P. aeruginosa* was enumerated using both the spread plate and membrane filtration methods on cetrimide agar, according to Standard Methods (D5465–16)^21^. Membrane filtration assays were performed using the entirety of sample solutions after volumes were removed for spread plating assays (~ 17.7 mL). MS2 was propagated and enumerated using the double-layer agar method on tryptic soy agar (TSA) with *Escherichia coli* 3000 (ATCC 15597) as a host, according to Method 1601^22^. Both *P. aeruginosa* and MS2 agar plate assays were incubated for 18–24 h at 37 °C.

### FFR coupon inoculation, exposure, and recovery

Coupons were made from 1-cm diameter material from 9210 and 8110 s or 8210 (equivalents) N95 FFRs (3 M, USA). N95 FFR strap coupons (1 cm^2^) were cut from 1860 and 8110 s/8210 N95 FFRs (3 M, USA). Before inoculation, *P. aeruginosa* or MS2 working stocks were diluted into either phosphate buffer solution (PBS) or TSB to the desired concentration. In the single case when an extremely high *P. aeruginosa* concentration was required, the working stock was centrifuged at 3,000 RPM for 10 min and resuspended in TSB. Depending on the experiment, duplicate or triplicate respirator material coupons or strap sections were placed inside sterile 47-mm Petri dishes and inoculated with the diluted *P. aeruginosa* or MS2 by pipetting 5-µL droplets onto the surface of the coupon. Droplets were then spread with a cooled flame-sterilized glass spreader and allowed to dry for 20 min. The estimated loadings are reported as colony-forming units (CFU) or plaque-forming units (PFU) per cm^2^ (Table 1).

**Table 1:** Experimental summary of organism, inoculation concentration, FFR material, exposure arrangement, fluences, and replicates.

Objective	Organism	Inoculation concentration (CFU/PFU cm^−2^)	FFR material	Layer-exposure arrangement (Fig. 2)	UV-C fluences (mJ cm^−2^)	Biological replicates
Fluence response (coupon)	MS2	6.9E + 08	9210	1 & 8	0, 50, 100, 500, 700, 1000	2 per fluence per arrangement
	MS2	6.9E + 08	8110 s/8210	1 & 8	0, 100, 500, 1000	2 per fluence per arrangement
	*P. aeruginosa*	3.9E + 06	9210	1	0, 100, 500, 1000	3 per fluence
Fluence response (straps)	MS2	6.9E + 08	9210 (rubber)	Direct	0, 50, 100, 500, 700, 1000	2 per fluence
			1860 (polyester)	Direct	0, 50, 100, 500, 700, 1000, 1400*	2 per fluence
Bacterial loading (coupon)	*P. aeruginosa*	4.0E + 03 4.0E + 04 3.2E + 05 1.6E + 06	9210	1	0 and 50	3 per fluence per concentration
		1.6E + 06 5.0E + 07	9210	1	0 and 1000	3 per fluence per concentration
Inter-layer inoculation	MS2	6.9E + 08	9210	1–8	0 and 500	2 per fluence per arrangement

*The polyester elastic absorbed the inoculation liquid; therefore, the elastic was flipped over after 700 mJ cm^−2^ to expose both sides.

Petri dishes containing inoculated coupon samples were placed 1.5 cm beneath the collimator onto a 30 RPM rotating platform with the inoculated surface facing up to ensure uniform UV exposure. Following exposure, each FFR coupon was placed in a sterile 50-mL conical tube containing 20 mL sterile PBS using flame-sterilized tweezers. Each strap coupon was placed in a sterile 2-mL dilution tube containing 0.9 mL sterile PBS. For both sample types, tubes containing samples were vortexed at 3000 RPM for 1 min to facilitate the shedding of microorganisms. The resulting liquid suspension from each sample, referred to as sample solution, was then used for serial dilutions or plated directly from the sample solution. For both spread plating and the double-layer agar method, serially diluted 100-µL samples were plated. In cases where high inactivation levels were anticipated, 1 mL of undiluted MS2 and *P. aeruginosa* sample solutions were plated.

Additionally, *P. aeruginosa* sample solutions were used for membrane filtration plating of *P. aeruginosa*. For each variable parameter (*i.e*., concentration, respirator material), positive controls (no UV exposure) were prepared for comparison with treated samples for log reduction value (LRV) estimation. Positive control recovery was carried out as described above, absent UV exposure.

### UV collimated beam apparatus

UV inactivation experiments were conducted using the 280 nm wavelength on a UV-C LED collimated beam apparatus (PearlLab Beam T 255/280/365, Aquisense, USA). Irradiance was measured using a USB4000 Ocean Optics spectroradiometer (Ocean Optics, USA). Irradiance measurements were collected with 0.5-mm spatial resolution across the face of the coupons and were used to calculate the Petri Factor (0.890). The average irradiance delivered to FFR coupons was determined using the product of a central irradiance measurement and the Petri Factor and was calculated to be 794 µW cm^−2^. The desired fluence was divided by the average irradiance to determine the required exposure time. It is important to note that the collimated beam setup coupled with coupons is an idealized arrangement and does not fully represent the use case of disinfecting FFRs within a hospital. Full FFR disinfection would involve 360° irradiance from a UV-C source and would necessitate the use of multi angle spectroradiometers to properly interpret an achieved fluence. Although there are limitations with the setup used in this work, it does provide a reproduceable approximation for understanding the dynamics of UV-C irradiation on FFR layer material.

### Intra-layer inoculation

**Figure 1:**
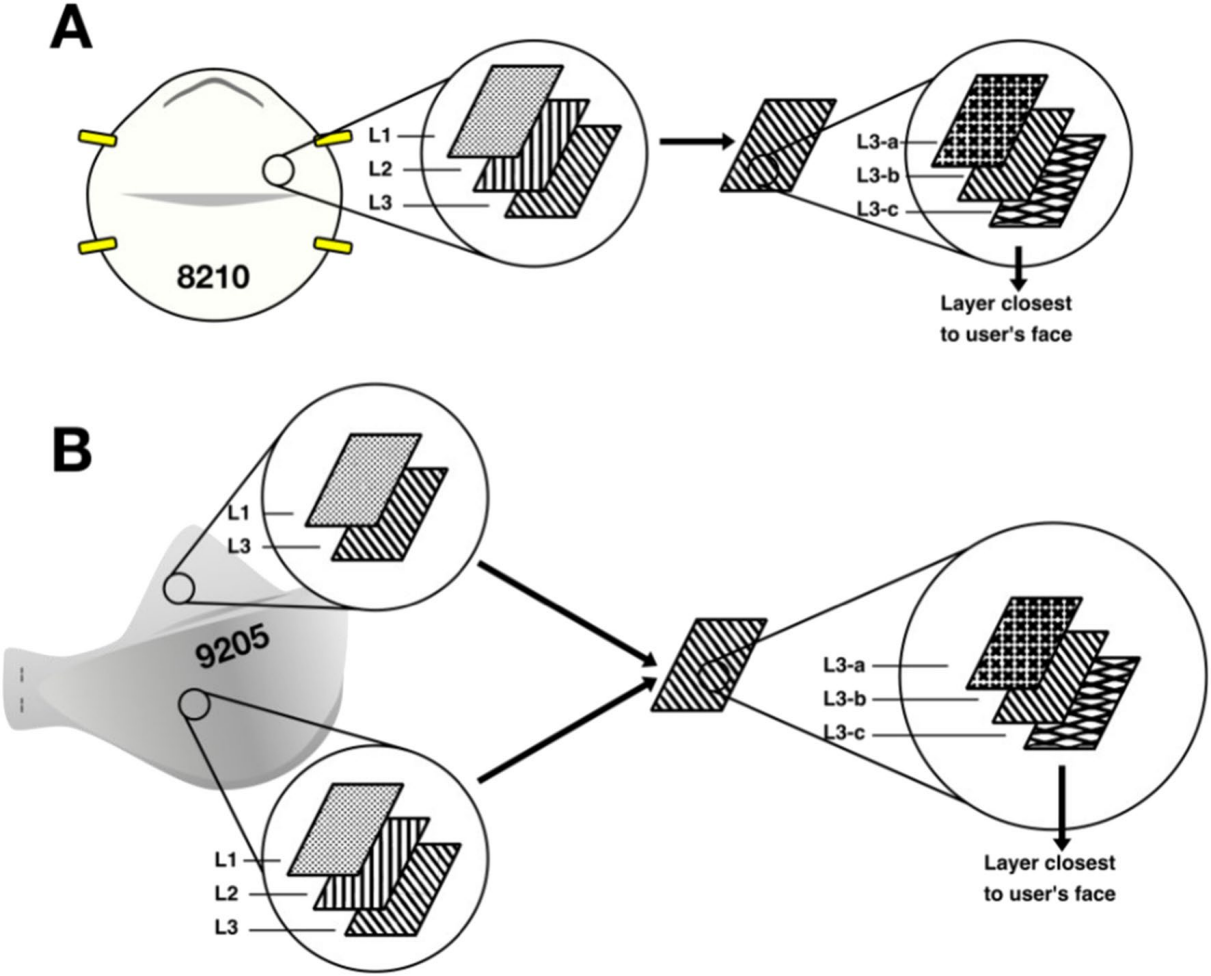
(**a**) N95 8210 layer arrangement, (**b**) N95 9210 layer arrangement. Note that side sections of the 9210 respirators lack an L2 layer.

N95 respirators are composed of multiple layers. The 9210 FFR (Fig. 1b) is composed of an outer hydrophobic layer (L1), a middle electrostatically charged layer (L2), and an inner biocompatible layer (L3), as described by a previous study^17^. L3 was dissected into three sub-layers (a, b, and c) to investigate the nature of the respirator material further. The hydrophobic nature of N95 respirator material is intended to repel 95% of aerosolized droplets; however, droplet nuclei may penetrate the outer hydrophobic layer and become trapped in the electrostatic layers of the material. An inter-layer study was conducted using coupons cut from the N95-9210 FFR model to investigate differences in the reduction of organisms that may be present within FFR layers. Eight combinations of inter-layer inoculation and UV-C exposure direction were investigated. Figure 1 illustrates the layer configuration and labelling scheme. All arrangements were exposed to 500 mJ cm^−2^ of UV-C 280 nm.

**Figure 2:**
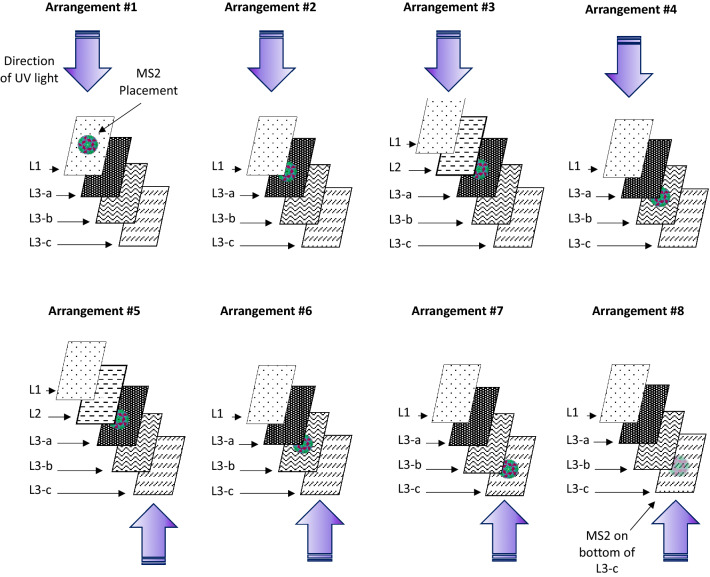
Inter-layer inoculation and UV exposure arrangements. Arrangements 1, 2, 4, and 6–8 represent the upper section of the 9210 FFR (comprised of L1 and L3). Arrangements 3 and 5 represent the front of the FFR (comprised of L1, L2, and L3).

Coupon layers were peeled back using flame-sterilized tweezers and inoculated as described in Sect. 2.4. Figure 2 illustrates the eight combinations of inter-layer inoculation location and exposure direction.

### Respirator layer analysis

**Figure 3:**
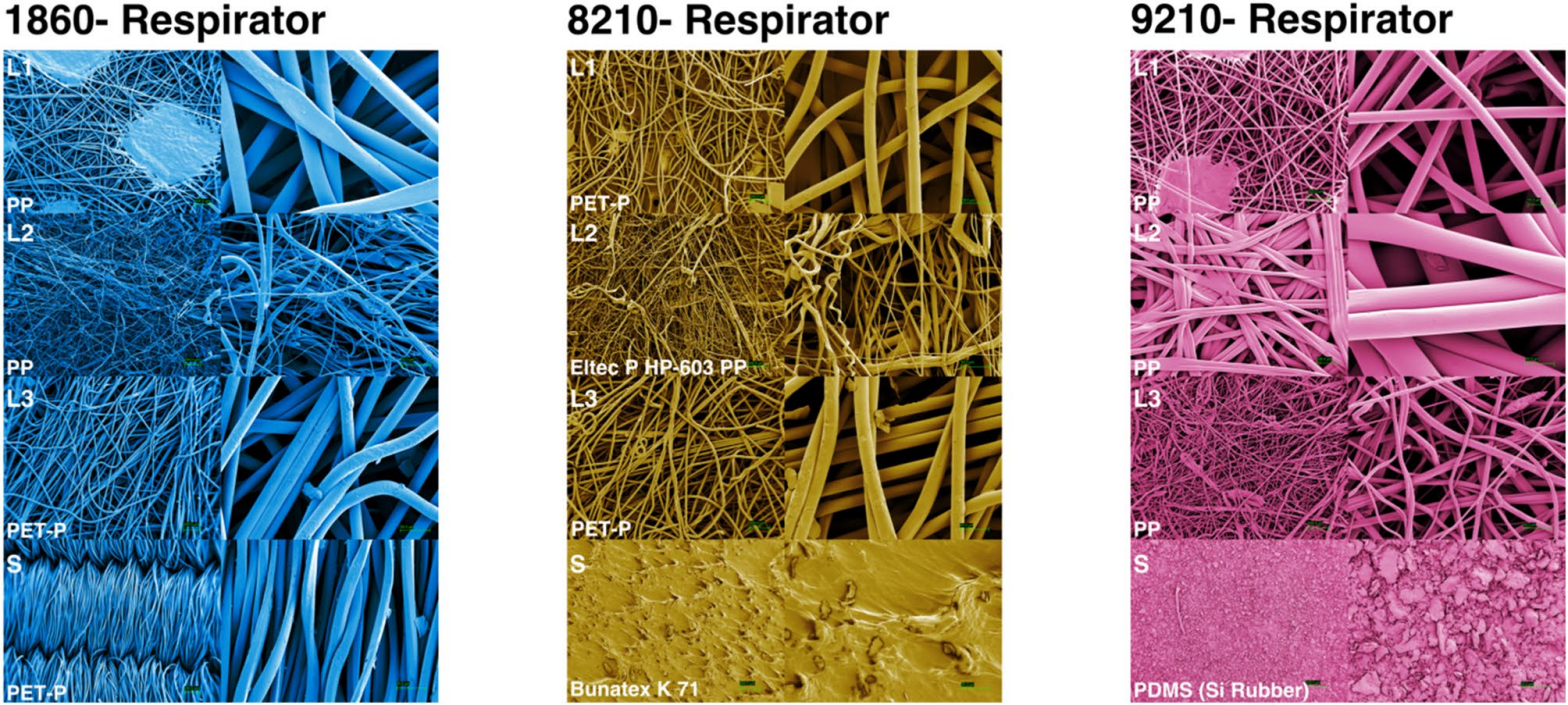
Material characterization summary of the 1860, 8210 and 9210 N95 respirators. The left columns of SEM images for each respirator model are ×55 magnification, and the right columns are ×302 magnification. SEM images are falsely coloured for clarity purposes.

Material characterization was conducted on different respirator models to analyze the different respirator layers and their role in disinfection efficacy. Layers and straps of N95 respirator models 9205, 9210, 8210, and 1860 were characterized using Fourier-transform infrared spectroscopy (FTIR) and scanning electron microscopy (SEM). Samples of FFR layers and straps from each of the models were treated with gold sputtering and then scanned at 302 and 55× magnification. FTIR measures the infrared absorption and emission spectra of the sample and infers the functional groups present. An FTIR (Bruker Alpha, USA) equipped with the solid-phase attachment was used for all samples. For FTIR analysis, only N95 1860, 8210 and 9210 models are presented in Fig. 3. Material characterization information for additional respirator models can be found in Supplementary Figure S2. FTIR samples were prepared by cutting a 1-cm^2^ coupon from each respirator layer and placing them beneath the FTIR instrument head. Sample spectra were matched with profiles from the Bruker library to identify the most probable material type. Spectra were then exported as CSV files for further data management using the R programming environment. Results can be found in Supplementary Figure S3.

### Irradiance through 9210 FFR layers

UV light penetration through the N95 9210 respirator layers was tested using the UV-C LED collimated beam apparatus (PearlLab Beam T 255/280/365, Aquisense, USA). The UV light was measured using a USB4000 spectrometer (Ocean Optics Inc., USA) to determine the proportion of UV light blocked by the layers of the 9210 respirators. Light penetration was assessed by clipping combinations of coupons of 9210 respirator layers to the surface of a spectroradiometer detector as described in a previous study^17^.

### Replicates, limits of detection and quantification, statistics, and data visualization

For each experiment, a minimum of two biological replicates (*i.e.,* two coupons) was performed for each combination of variables (*i.e.,* fluence, respirator layer). Each biological replicate was enumerated via two technical replicates (*i.e.,* two assays) across a minimum of three tenfold dilutions for spread-plating (*P. aeruginosa*) and the double-layer agar (MS2) assays. A single membrane filtration assay was performed for each biological replicate of treated *P. aeruginosa* samples. Positive controls were processed for each experiment, including separate sets of controls when different materials, layers, or concentrations were investigated. All data were analyzed and visualized using R v4.0.0 and RStudio v1.1.463^23^ using the Tidyverse^24^, Ggplot2^25^, Dplyr^26^, Ggsci^27^ and RColorBrewer^28^ packages. For both *P. aeruginosa* and MS2, non-detects (ND) were < 1 CFU/PFU per maximum volume plated. Where appropriate, paired t-tests were conducted at a significance level of α = 0.05. When comparing UV fluences, one-sided t-tests were performed. In all other cases, two-sided t-tests were performed.

### Experimental design

A fluence of 50 mJ cm^−2^ was used to test the UV-C efficacy of four *P. aeruginosa* loading concentrations. Preliminary experimentation (data not shown) suggested that in some instances, 100 mJ cm^−2^ was sufficient to reduce *P. aeruginosa* to below detection; therefore, a fluence of 50 mJ cm^−2^ was chosen to measure disparities in LRVs between different loading concentrations. The loading concentrations are described in Table 1.

UV-C (280 nm) light blockage was investigated using various layer combinations for both the 9210 and 8110 s N95 models. Respirator layers were fastened on top of the spectrometer, then placed below the collimated beam. Thirty irradiation readings were taken per layer combination and averaged. These values were then compared to the averaged readings taken when no layers were present between the UV-C light source and spectrometer to estimate the percentage of 280 nm light blocked by each layer or layer combination.

For clarity, a summary of all experiments is detailed in Table 1, which includes the objective, test-organism, layer-exposure arrangement (see Fig. 2), fluences, coupon/elastic material, biological replicates, and microorganism loading.

### Control experiments

Since it is not common to combine spread plating and membrane filtration for enumeration comparison, as they may vary in accuracy and precision, a recovery experiment was performed to determine if there was a significant difference in recovery efficiency between the two methods. To do so, *P. aeruginosa* was assayed via both enumeration techniques with 12 replicates for both. The results of these experiments can be found in SI 1.

## Results and discussion

### Effects of respirator material and UV-C fluence

**Figure 4:**
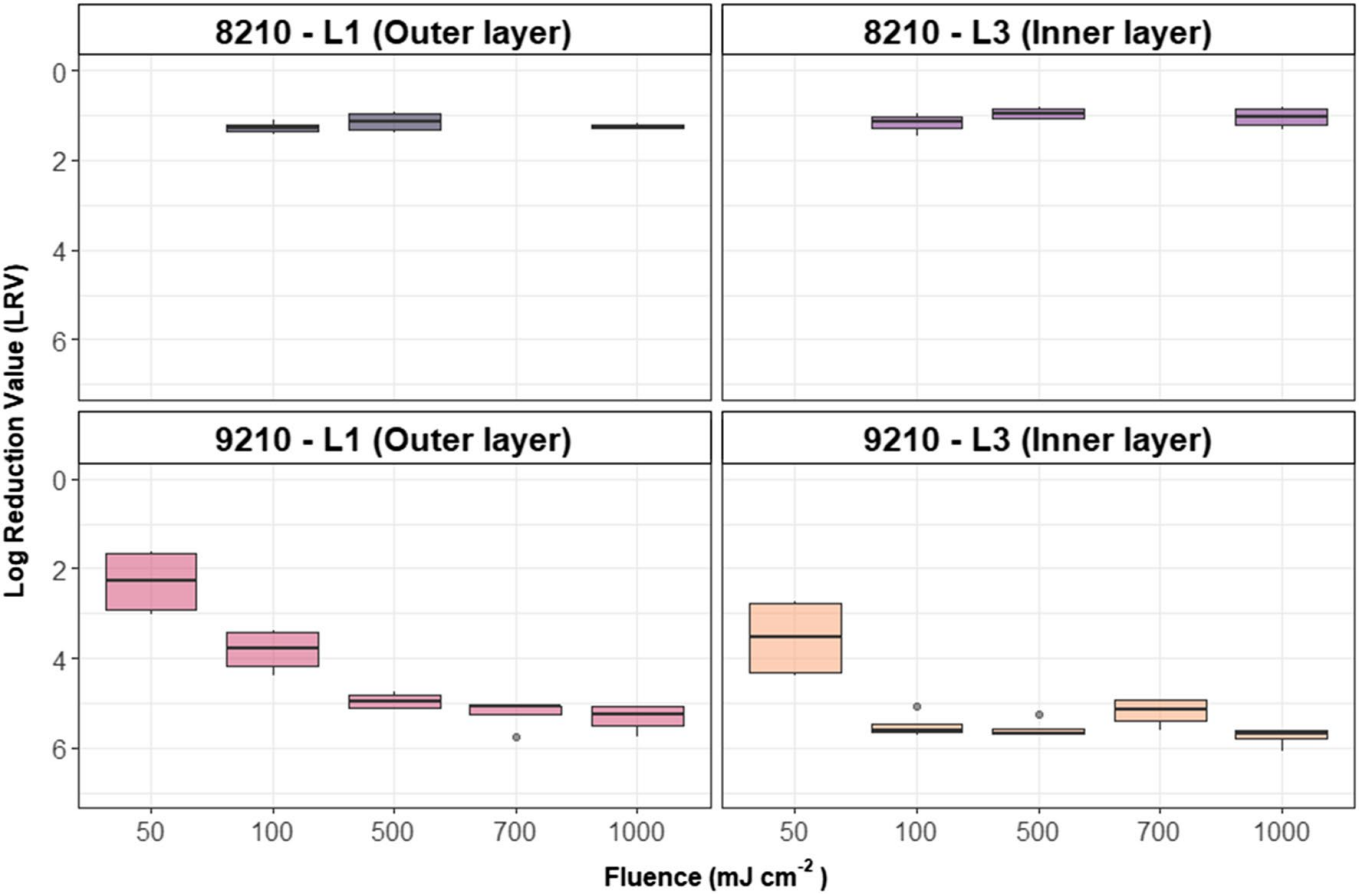
MS2 fluence–response curve for four combinations of respirator-type and respirator layers (Fig. 1a,b) with UV-C 280 nm irradiation. Each box represents each of 2 technical replicates for each of 2 biological replicates for a total of 4 data points. An outlying dot indicates data points that are 1.5 * IQR (interquartile range) outside the first or third quartiles.

Coupons from two 3 M N95 respirator models (8110 s and 9210) were inoculated with MS2 and exposed to an array of UV-C 280 nm fluences ranging from 50 to 1000 mJ cm^−2^ to examine the relative effects of fluence and type of respirator material. The results of this experiment are shown in Fig. 4.

#### FFR model 9210

At 50, 100, and 500 mJ cm^−2^ (Fig. 4), MS2 LRVs observed for the 9210 FFR differed significantly between L1 and L3 (*p* = 0.0009, 0.01, and 0.01, respectively). At fluences of 700 and 1000 mJ cm^−2^, no significant difference in MS2 reduction was observed between L1 and L3 (*p* = 0.9 and 0.07, respectively). This difference suggests that the outer-most layer (L1) requires a higher UV-C fluence for inactivation. For L1, MS2 LRV increases between fluences were significant up until 500 mJ cm^−2^, after which no significant differences were observed. For L3, LRV increases were significant between 50 and 100 mJ cm^−2^ (*p* = 0.02), after which no significant increases in LRV were observed. In Supplementary Figure S1, *P. aeruginosa* LRVs observed for 9210-L1 were similar to that observed with MS2. The similarity in kinetics between MS2 and *P. aeruginosa* was surprising, given that MS2 typically requires UV fluences which are orders of magnitude higher than what is required for a similar LRV of *P. aeruginosa*^29,30^. Supplementary Figure S1 illustrates an observable difference in *P. aeruginosa* LRVs between 100 and 500 mJ cm^−2^. Between 500 and 1000 mJ cm^−2^, the LRVs were virtually the same, both having two treated samples with non-detectable levels of *P. aeruginosa*.

### FFR model 8110 s/8210

A significant difference in LRV was observed between L1 and L3 of the 8110 s FFR at 500 mJ cm^−2^ (*p* = 0.02) but not at 100 and 1000 mJ cm^−2^ (*p* = 0.3 for both fluences). Further, there was no significant increase in LRV at fluences after 100 mJ cm^−2^ (*p* > 0.05). The most drastic LRV differences were observed between the 9210 and 8110 s respirator models. Significant differences (*p* << 0.05) were observed between both respirator models for both arrangements and all paired fluences (100, 500, and 1000 mJ cm^−2^).

The disparities in LRV observed between the two respirator models are consistent with previous studies^5,31^, who found that the effectiveness of various decontamination methods was model-dependent, given their differences in design, materials, and hydrophobicity. However, a more recent study^32^ did not find a statistical difference between hydrophobic versus hydrophilic respirator materials. In summary, the outer-most layer (L1) of the 9210 FFR requires higher UV-C fluences than the inner-most layer (L3) to reach the maximum observed MS2 LRV of just over 5. Additionally, UV is considerably more effective for inactivating MS2 on 9210 FFRs relative to 8110 s FFRs and higher than 5 LRVs on MS2 are achievable at UV-C 280 nm fluences of 100 and 500 mJ cm^−2^ for L3 and L1 of the 9210 FFR, respectively.

As shown in Fig. 4, both respirator models and the inoculation/exposure arrangement considerably affected UV efficacy. Although both FFR models are comparable in terms of certification by The National Institute for Occupational Safety and Health (NIOSH), they responded differently in terms of UV disinfection. LRVs above 1.5 were not observed with the 8110 s/8210 FFR model, even at fluences of 1000 mJ cm^−2^. The 9210 FFR model resulted in a more pronounced disinfection curve, where LRVs between 5 and 6 were observed for both L1 and L3 starting at doses of 500 and 100 mJ cm^−2^, respectively. Proposed stringent regulations of 3 and 6 LRVs^33^ for Tier 3 and Tier 2 certification, respectively; however, these results suggest that UV disinfection technologies may behold specificity for FFR models, which is not contemplated in regulatory constructs. Future UV validation studies for FFR disinfection should place emphasis on the model of FFR investigated to understand disinfection efficacy and specificity further.

### FFR elastic material and UV-C fluence

Strap segments from two FFR models were inoculated with MS2 and exposed to UV-C irradiation at fluences of 280-nm. The objectives were to (1) understand the UV-C kinetics for MS2 on FFR straps and (2) determine if strap material plays a role in UV efficacy for treating FFR straps. Rubber-elastic (9210) and polyester-elastic (1860) straps were tested. The LRVs observed with the rubber elastic from the 9210 FFR increased steadily until 500 mJ cm^−2^, where all samples were reduced to either below the LOQ or below detection. However, the LRVs observed with the polyester elastic from the 1860 FFR were considerably less. LRVs remained at or below 0.5 for fluences of 50 to 1000 mJ cm^−2^. When each side of the elastic was exposed to 700 mJ cm^−2^ for a total of 1400 mJ cm^−2^, LRVs were higher than when one side was exposed to 1000 mJ cm^−2^; however, the difference was relatively small (0.17, *p* = 0.003).

**Figure 5:**
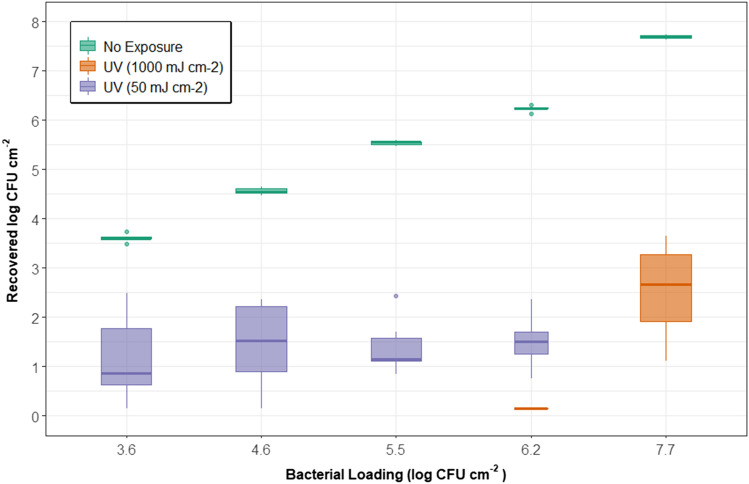
Relative effects of *P. aeruginosa* cell-density on 9210 FFR coupon LRVs. Each boxplot represents each of 2 technical replicates for each of 2 biological replicates.

Figure 5 shows that similarly to the 9210-respirator material, MS2 LRVs > 5 are observed at a fluence of 500 mJ cm^−2^ for 9210 rubber-elastic straps. When treating the polyester-elastic 1860 respirator straps, it was impossible to achieve more than one LRV, even when exposed to UV-C fluences higher than 1000 mJ cm^−2^, which are generally sufficient under other conditions. Additionally, even when the 1860 polyester straps were exposed to UV-C fluences (700 mJ cm^−2^ per side), LRVs were not substantially increased. SEM results (Fig. 3) show that the polyester strap is much more intricate at a microscopic level than the rubber strap from other FFR models. This intricate geometry is likely absorbing the inoculum and preventing UV-C radiation from penetrating the strap material, in contrast with rubber straps where the inoculum droplets stay on top of the material.

Including strap material in disinfection experiments is critical to determine if specific N95 FFRs models will be suitable candidates for a given disinfection strategy. The results presented in Fig. 5 show that the 1860 FFR model and any other model with polyester nature straps may not be suitable for UV-C disinfection, at least below an applied fluence of 1400 mJ cm^−2^.

### Effects of bacterial loading

The relative effects of microbial concentrations on disinfection performance are not well understood, especially concerning FFR materials. For this reason, we investigated the relative effects of *P. aeruginosa* cell-density inoculated onto 9210 FFR coupons on LRVs (Fig. 3).

*P. aeruginosa* was recovered at similar concentrations in treated samples (*p* < 0.05 for all comparisons) across all cell-densities examined, resulting in an increased LRV as cell-densities increased (Fig. 3). Increased cell-densities that would result in more significant shielding were expected but were not observed in this result. The data in this study suggest that if cell-densities are too low, there is a diminishing return in efficacy with respect to LRVs. The implications of these results are essential for the design of standardized performance-testing protocols. Furthermore, a more direct comparison between studies would be possible if cell-densities were reported. For example, a recent study^32^ achieved > 5 LRV with MS2, whereas other studies^13,34^only achieved around 3 LRV with MS2 as well. None of these studies mentioned cell density; additionally, inoculation medium, FFR model used, and inoculation technique was also different. These disparity among studies may be impacted by cell-densities or other factors that were different, such as respirator type and inoculation location, as shown in Fig. 4.

A second experiment was conducted to address potential differences at higher cell-densities and a higher UV-C 280-nm fluence. As shown in Fig. 5, there may exist a critical cell-density threshold somewhere between 6.2 and 7.7 log CFU cm^−2^, in which cell-densities exceeding such a threshold result in over-aggregation of bacterial cells, leading to reduced LRVs.

### Materials characterization of three N95 FFR models

Figure 3 summarizes the material characterization of the 1860, 8210 and 9210 N95 respirators. Only the three primary and more easily separated layers were analyzed. Layer nomenclature is as depicted in Fig. 1a. The main polymers found in the layers of the respirator were Polypropylene (PP), Polyethylene Terephthalate (PET-P), Polydimethylsiloxane (PDMS), and Eltec P HP-603 Polypropylene.

PP is considered a thermoplastic polymer used in a wide range of applications. PP presents non-polar properties, which indicates a low interaction with water^35^. In contrast, PET is a polar plastic, commonly found in plastic bottles^36^. PET has an intrinsic viscosity and hygroscopic nature (retains water from its surroundings). Moreover, PDMS is a commercially-available silicon rubber^37^ that is viscoelastic and hydrophobic (repels water).

The 1860 model layers were mainly comprised of PP (L1 and L2) and PET (L3), while the straps were composed of PET. Comparatively, the 9210 model mainly had PP (L1, L2 and L3) and PDMS (rubber) for the straps, while the 8210 model primarily consisted of PET (L1 and L3), Eltec P HP-603 Polypropylene (L2), and Bunatex K 71 (straps).

**Figure 6:**
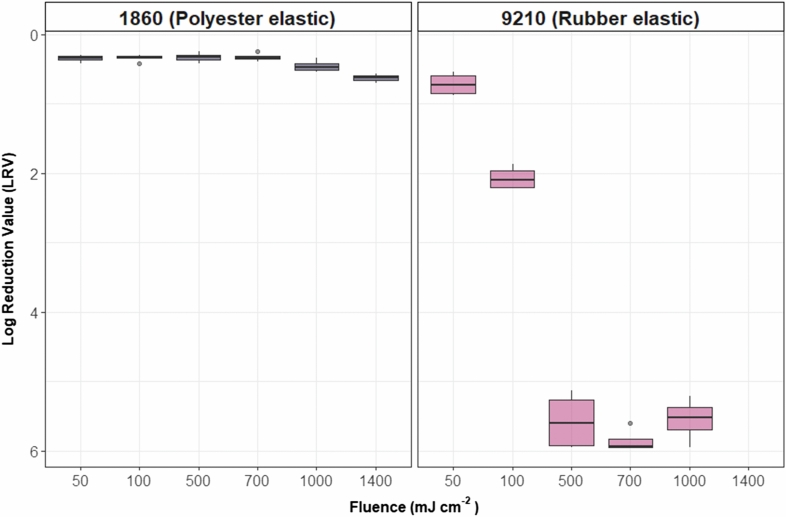
MS2 fluence-response curve for two FFR elastic types with UV-C 280 nm irradiation. *Note*: for the polyester elastics exposed to 1400 mJ cm^−2^, each side was exposed to a fluence of 700 mJ cm^−2^. Each point represents each of 2 technical replicates for each of 2 biological replicates.

The respirator materials and their configuration within the layers of the respirators could explain the difference in disinfection found in the respirator microbial testing. Figures 4 and Fig. 6 show that the 9210 respirator achieved higher LRVs in both MS2 and *P. aeruginosa* tests.

The 1860 respirator straps, which have a fabric-like texture, resulted in low LRVs compared to the 9210 model, even when applying 700 mJ cm^−2^ per side. The low LRV value achieved on the 1860 strap may be attributed to the hygroscopic nature of PET, contrary to the 9210-respirator strap, which is mainly composed of hydrophobic PDMS.

The higher disinfection efficiency on the 9210-respirator strap, compared to the 1860-respirator strap, may be attributed to the hydrophobic nature of the strap. Hydrophobic layers ensure that the bulk of UV exposure occurs on the surface of respirators. In contrast, hygroscopic layers enhance the penetration of inoculum deeper into the textile, inhibiting the microorganisms from being adequately exposed to UV light. Figure 3 shows the overall structure for each of the tested respirator layers and depicts gaps in the material where droplets could reach. It is worth noting that since FFRs are single-use PPE items, the original design of the straps and choice of material was likely based on other desired features, such as comfort and durability. Incidentally, the pandemic has created a unique demand on the repurposing of FFRs (REF), and the strap material must be assessed in disinfection experiments as it appears to have a profound effect on UV disinfection efficiency.

The findings presented in this manuscript provide evidence that respirators with hygroscopic properties may not be suitable for UV disinfection alone, as their absorbent materials may attract and retain droplets where microorganisms are present. Other studies have also found that different FFR models respond differently to UV disinfection^13,32^. However, comparison between FFR models and their material properties has not often been incorporated in previous studies, or their results have been inconclusive^38,39^.

### Implications of UV disinfection on surfaces

UV disinfection has been used primarily in the drinking water and wastewater industries since the maturation of the technology in the past decades^40^. More recently, UV technology has been increasingly used for disinfection of surfaces in hospital settings^41–43^. A recent study examined the impact of common hospital surfaces (plastic, stainless steel and copper) on UV disinfection efficiency^44^; however, there is still a significant gap in UV surface disinfection knowledge. As mentioned by a recent study^45^, the applied UV fluence delivered onto a surface does not necessarily reflect the UV fluence received. Moreover, surface irregularities and crevices at the microscopic level could create shadowed areas where the UV light cannot penetrate. Furthermore, the porous multilayer structure of an N95 FFR requires roughly two orders of magnitude higher applied UV fluence for sufficient inactivation when compared to a smooth surface material.

The COVID-19 pandemic has created an urgent need and interest to disinfect a broader range of complex surfaces, including N95 FFRs, surgical masks and other forms of PPE. This scenario presents a challenge for the development of disinfection protocols as there are many factors that can influence the effectiveness of UV treatment, such as surface geometry, material type and FFR construction. Recent studies have successfully applied UV technology for the repurposing of PPE in clinical settings^39,46^; moreover, systematic reviews on the topic have concluded that UV disinfection is a suitable technology for PPE repurposing^12,45,47–49^. However, not all studies have considered the effect of different FFR layer materials on UV disinfection efficacy.

While the current literature agrees that UV disinfection is suitable for FFR repurposing, there has not been a consensus on the UV fluence required. However, an application of at least 1000 mJ cm^−2^ is the most common value reported^13,31,46^. Additionally, there is not unanimity on the required LRV to claim successful FFR disinfection, as these values have ranged from > 3 to > 6 LRV and involved different target microorganisms. Moreover, there is still debate whether the type of FFR material dictates the UV fluence required for disinfection, even though some studies have found evidence that the hydrophobicity/hydrophilicity of materials play a role in disinfection efficacy^13,31,50^. In contrast, other study^32^did not find a difference in disinfection performance between hydrophilic and hydrophobic materials used in FFR layers; however, the authors of the study did not provide a characterization of the materials.

The inconsistency of results between studies for UV disinfection of FFRs could be attributed to the exclusion of the impact of respirator materials on disinfection performance. This work indicates that not all materials used in the construction of FFRs respond equally to UV treatment. To the author’s knowledge, this is the first manuscript that analyzes FFR disinfection efficiency as a function of layer material and composition when using a UV-C light source at 280 nm. It is hypothesized that differences in disinfection efficiency will be similarly impacted by material type across the UV-C spectrum.

### Respirator layer analysis

**Figure 7:**
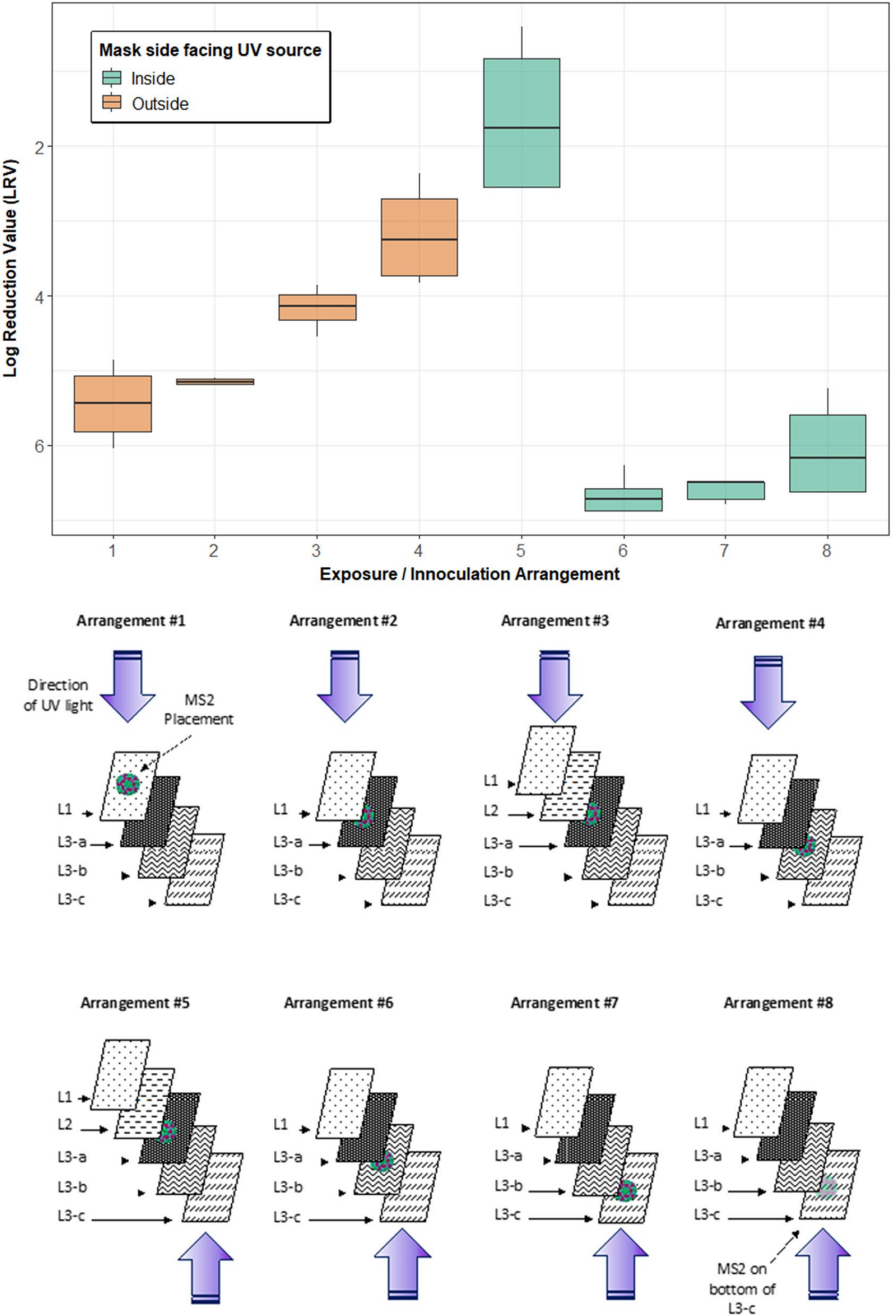
UV-C 280 nm exposure of various inter-layer inoculation combinations and exposure direction.

N95 FFRs are designed to repel droplets from the outer layers and electrostatically trap microorganisms within the respirator’s inner layers^51^. However, the reuse of N95 FFRs may still pose a health hazard to users if pathogenic microorganisms are not adequately inactivated. An experiment was conducted to assess several combinations of inter-layer MS2 inoculation and UV-C exposure direction at a fluence of 500 mJ cm^−2^. Figure 7 shows the differences in LRVs achieved.

MS2 recoveries for 9210 FFR layers varied layer to layer, which impacted the level of measurable LRVs in many cases. Arrangements 3 and 5 were the only arrangements that used the front section of the 9210 FFR, which included L2 (Fig. 1). Tests with all other arrangements were carried out with the top section of the 9210 FFR, which did not include L2.

The high LRVs for arrangements where embedded layers were inoculated were L3-a, depicted in Fig. 1, was decisively the layer that blocked the most UV-C light. This can be concluded by the fact that arrangements 4 and 5 were the only arrangements that did not result in LRVs greater than 4. These results suggest that if 9210 FFRs are exposed to UV-C 280 nm from both sides, LRVs above 4 may be expected at fluences of 500 mJ cm^−2^. However, this may be a best-case scenario and does not account for areas on the respirator where additional blockage may occur, such as straps and nose pads.

## Conclusions

Filter facepiece respirators (FFR) models are comprised of different materials and numbers of layers; therefore, FFR models yield different log-removal values following UV treatment. The respirator layer that was selected for inoculation also impacted the disinfection efficacy of UV-C exposure. Additionally, the variability in FFR design invalidates a universal treatment approach for disinfection. Some respirator models may be suitable for decontamination and reuse using low levels of UV fluence, while other models need a much higher amount of irradiation to overcome material properties that inhibit UV exposure. Another important observation from this work was that not all sections of the respirator responded to UV treatment equally. The variety and complexity of materials used in the construction of FFRs results in a challenging surface to disinfect; for example, the straps of the 1860 model were highly resistant to disinfection (due to its intricate and hygroscopic PET nature), while higher LRVs were observed with the PDMS straps present on the 9210 FFR.

A standardized protocol (in terms of inoculation volume, placement, cell-density, inoculum and FFR material) and reporting methodology for these results are also required to ensure safe and replicable decontamination. The authors recommend that the points mentioned in this paper are taken into consideration when designing testing and validation experiments using UV technology for FFR decontamination. Through this work, it is evident that the difference in material surface of N95 respirators will result in significant differences in UV disinfection efficacy and to maximize user safety, it is likely that only specific N95 respirators may be used for UV disinfection. The results presented in this study have implications for UV disinfection of materials, which extends to many possible applications well beyond respirator decontamination. Considering the high interest of surface disinfection, the results presented in this manuscript help the development of standardized protocols for the disinfection of complex materials using UV technology.

## Supplementary Information

Supplementary Information.

## Data Availability

The datasets generated during and/or analyzed during the current study are available from the corresponding author on reasonable request.
