# Supplementary material for: Specificity of UV-C LED disinfection efficacy for three N95 respirators
Source: Sci Rep. 2021 Jul 28;11:15350. doi: 10.1038/s41598-021-94810-4 (PMC8319424; doi:10.1038/s41598-021-94810-4)
Supplement: Supplementary file 1 — Supplementary Information. [file 41598_2021_94810_MOESM1_ESM.docx]

**SUPPLEMENTARY INFORMATION**

**Title**

Specificity of UV-C LED disinfection efficacy for three N95 respirators

**Authors**

C. Carolina Ontiveros¹, David Shoults¹, Sean MacIsaac¹, Kyle D. Rauch¹, Crystal L. Sweeney¹, Amina K. Stoddart¹, Graham A. Gagnon¹*.

¹ Centre for Water Resources and Studies, Department of Civil and Resource Engineering, Dalhousie University, 1360 Barrington St, Halifax, NS, B3H 4R2, Canada.

* Corresponding Author: Graham A. Gagnon

Email: [graham.gagnon@dal.ca](mailto:graham.gagnon@dal.ca)

Address: Department of Civil and Resource Engineering, Dalhousie University, 1360 Barrington St. Halifax, NS, Canada (B3H 4R2)

Phone: +1 902 494 6070

**SI 1. Paired spread plating and membrane filtration experiment.**

To address potential disparities in enumeration methods for *P. aeruginosa*, a paired experiment was performed. Both enumeration methods showed high precision. The mean log-transformed concentrations were 8.01 ± 0.06 and 8.10 ± 0.07 for spread plating and membrane filtration, respectively. The difference was statistically significant (p = 0.002). However, while statistically significant, it is important to consider how this difference impacts the conclusions that can be drawn from the experiments described in this study. Given that the concentrations measured by membraned filtration were slightly higher than that of spread plating, LRVs may be underestimated when comparing untreated samples measured by spread plating to treated samples measured by membrane filtration. Practically speaking, the log reduction story remains relatively unchanged, though, should be kept in mind.

**SI 2. Effects of respirator material and UV-C fluence (*P. aeruginosa*)**

Coupons from 3M N95 9210 respirator were inoculated with *P. aeruginosa* and exposed to UV-C 280 nm fluences of 100, 500 and 1,000 mJ cm^-2^ to examine the relative effects of fluence compared when compared to MS2 testing. The results of this experiment are shown in Supplemental Figure S1.


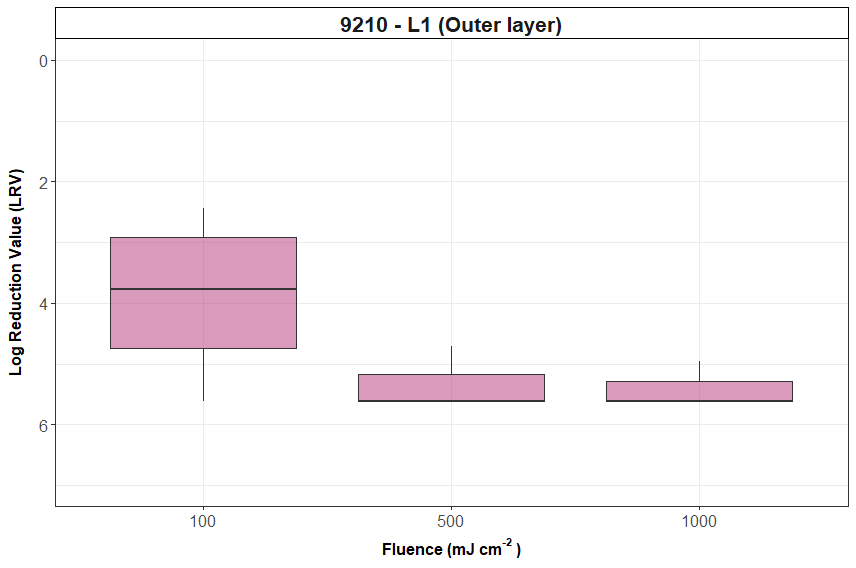


**Supplementary Figure S1**. *P. aeruginosa* fluence-response curve with UV-C 280 nm irradiation. Each box represents each of 2 technical replicates for each of 2 biological replicates for a total of 4 data points.

**SI 3. FTIR results**

**Supplementary Table S1**. Results from the FTIR on different FFR models. Layer nomenclature corresponds Figure 2 of main manuscript, S stands for straps.

| FFR Model | Layer | Material |
| --- | --- | --- |
| 9205 | L1 | Eltec P HP-603 Polypropylene |
|  | L2 | Eltec P HP-603 Polypropylene |
|  | L3 | Eltec P HP-603 Polypropylene |
|  | S | Silicone Rubber |
| 9210 | L1 | Polypropylene |
|  | L2 | Polypropylene |
|  | L3 | Polypropylene |
|  | S | Polydimethylsiloxane |
| 8210 | L1 | Polyethylene Terephthalate |
|  | L2 | Eltec P HP-603 Polypropylene |
|  | L3 | Polyethylene Terephthalate |
|  | S | Bunatex K 71 |
| 1860 | L1 | Polypropylene |
|  | L2 | Polypropylene |
|  | L3 | Polyethylene Terephthalate |
|  | S | Polyethylene Terephthalate |

**SI 4. SEM results (additional information).**

Supplemental Figure S2 presents the SEM results from the 9205 FFR model. These results were not presented in the main body of the manuscript, as there is no disinfection data paired with them.


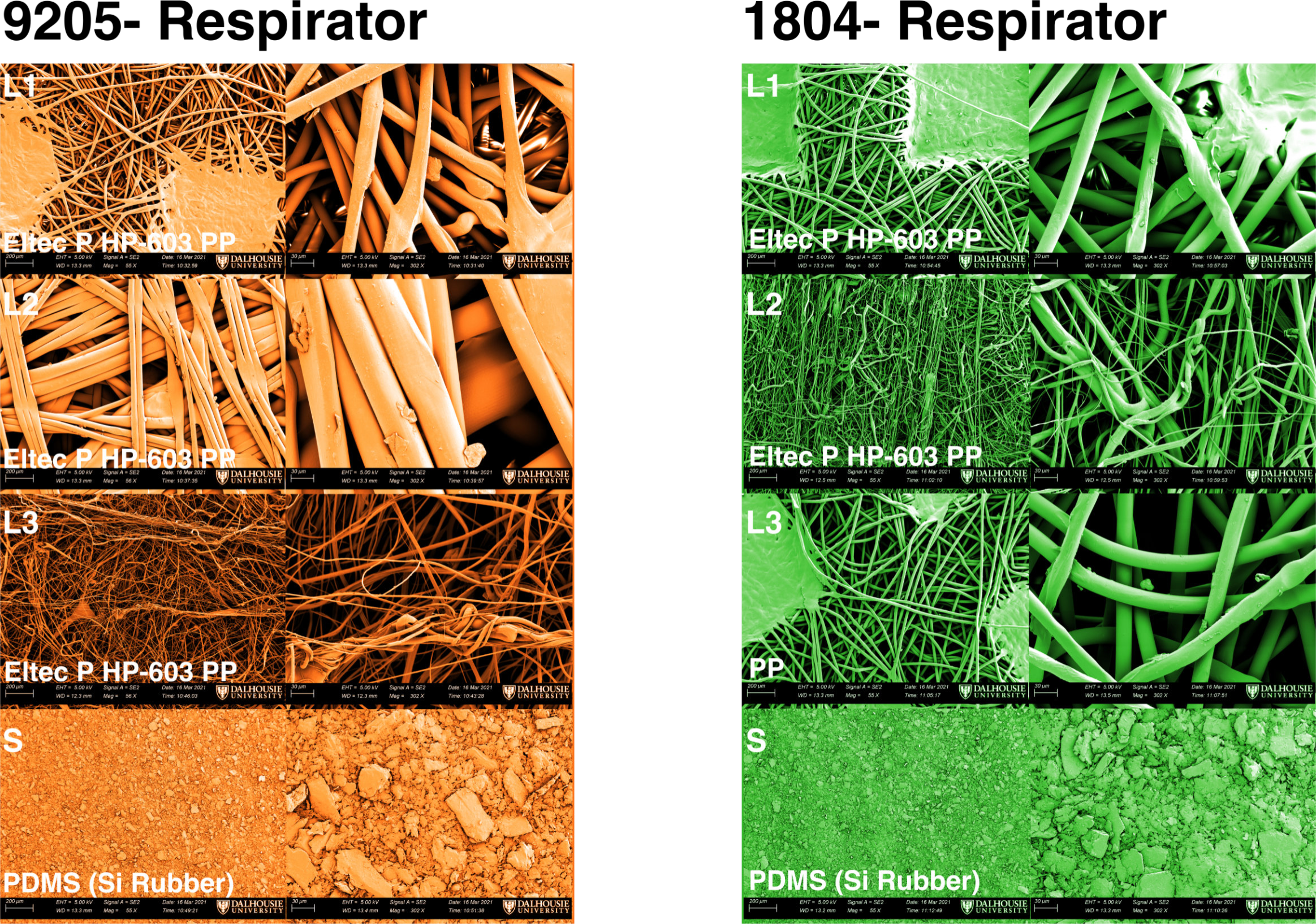


**Supplementary Figure S2**. Material characterization summary of the 9205 N95 respirators. Left columns of SEM images for each respirator model are 55x magnification and the right columns are 302x magnification. SEM images are false colored for clarity purposes.

**SI 5. FTIR spectra**

Supplemental Figure S4 shows FTIR spectra for five different FFRs models. Data was processed using R v4.0.0 and RStudio v1.1.463 ^22^, the Tidyverse ^23^, Ggplot2 ^24^, and RColorBrewer ^27^ packages.

**
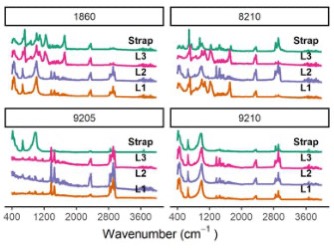
**

**Supplementary Figure S3**. FTIR data from five FFRs models. Layer nomenclatures correspond to Figure 2 from the main manuscript.
